# Inhibition of Hazara nairovirus replication by small interfering RNAs and their combination with ribavirin

**DOI:** 10.1186/1743-422X-8-249

**Published:** 2011-05-21

**Authors:** Olivier Flusin, Solenne Vigne, Christophe N Peyrefitte, Michèle Bouloy, Jean-Marc Crance, Frédéric Iseni

**Affiliations:** 1 Unité de virologie, Institut de Recherche Biomédicale des Armées (IRBA), 24 avenue des Maquis du Grésivaudan 38702 La Tronche, France; 2 Unité de Génétique Moléculaire des Bunyavirus, Institut Pasteur, 25 rue du docteur Roux 75724 Paris cedex 15, France; 3 Department of Pathology and Immunology, Centre Médical Universitaire, 1 rue Michel Servet 1211 Geneva 4, Switzerland

## Abstract

**Background:**

The genus *Nairovirus *in the family *Bunyaviridae *contains 34 tick-borne viruses classified into seven serogroups. Hazara virus (HAZV) belongs to the Crimean-Congo hemorrhagic fever (CCHF) serogroup that also includes CCHF virus (CCHFV) a major pathogen for humans. HAZV is an interesting model to study CCHFV due to a close serological and phylogenetical relationship and a classification which allows handling in a BSL2 laboratory. Nairoviruses are characterized by a tripartite negative-sense single stranded RNA genome (named L, M and S segments) that encode the RNA polymerase, the Gn-Gc glycoproteins and the nucleoprotein (NP), respectively. Currently, there are neither vaccines nor effective therapies for the treatment of any bunyavirus infection in humans. In this study we report, for the first time, the use of RNA interference (RNAi) as an approach to inhibit nairovirus replication.

**Results:**

Chemically synthesized siRNAs were designed to target the mRNA produced by the three genomic segments. We first demonstrated that the siRNAs targeting the NP mRNA displayed a stronger antiviral effect than those complementary to the L and M transcripts in A549 cells. We further characterized the two most efficient siRNAs showing, that the induced inhibition is specific and associated with a decrease in NP synthesis during HAZV infection. Furthermore, both siRNAs depicted an antiviral activity when used before and after HAZV infection. We next showed that HAZV was sensitive to ribavirin which is also known to inhibit CCHFV. Finally, we demonstrated the additive or synergistic antiviral effect of siRNAs used in combination with ribavirin.

**Conclusions:**

Our study highlights the interest of using RNAi (alone or in combination with ribavirin) to treat nairovirus infection. This approach has to be considered for the development of future antiviral compounds targeting CCHFV, the most pathogenic nairovirus.

## Background

Hazara virus (HAZV) is a member of the genus *Nairovirus *of the family *Bunyaviridae *which also includes *Orthobunyavirus*, *Hantavirus*, *Phlebovirus *and *Tospovirus*. *Nairovirus *comprises 34 tick-borne viruses classified into seven serogroups. The main representative serogroups are the Nairobi sheep disease group containing Nairobi sheep disease virus (NSDV) and Dugbe virus and the Crimean-Congo hemorrhagic fever (CCHF) group including HAZV and Crimean-Congo hemorrhagic fever virus (CCHFV) [1,2]. While NSDV induces acute hemorrhagic gastroenteritis in sheep and goats, CCHFV is responsible of severe hemorrhagic fever in humans associated with elevated levels of mortality (up to 50%) [1,3]. Due to its high pathogenicity for humans and because of the lack of therapeutics, CCHFV must be handled in BSL4 (biosafety level 4) laboratory [1]. Widely distributed throughout Eastern Europe, Asia and Africa, CCHFV represents a major public health problem [4,5] and is now considered as an emerging disease [6,7]. HAZV was isolated for the first time in 1954 from *Ixodes *ticks collected in Pakistan [8-10]. Although its natural host is not known, antibodies against HAZV were detected in rodent sera [11]. While non pathogenic for humans, it is lethal in new-born mice [12] and elicits cross-protection against CCHFV challenge in adult mice [13]. HAZV represents an alternative model to study CCHFV due to its close serological and phylogenetical relationship [13]. Furthermore, it can be handled in BSL2 laboratories.

The nairovirus are spherical enveloped particles of 100 nm in diameter. Their genome consists of three segments of single-stranded RNA of negative polarity designated S (Small), M (Medium) and L (Large) RNA segments. These three segments encode the nucleocapsid protein (NP), the envelope glycoproteins (Gn and Gc) and an RNA-dependent RNA polymerase (L), respectively [2,14,15]. During the viral cycle, NP and L drive the processes of transcription (mRNA synthesis) and replication (synthesis of genomic RNA) that occur in the cytoplasm [2]. Thus, targeting these proteins is likely an accurate strategy to inhibit the viral replication.

Currently, there are neither vaccines nor effective therapies to treat bunyavirus infection in humans. Ribavirin, however, has been shown to inhibit CCHFV replication in Vero cells [16], reducing the mean time to death in infant mice [17] and partially protecting infected STAT-1 KO mice [18]. Several studies reported the efficiency of oral or intravenous use of ribavirin to treat CCHFV infection cases [19-21] but to date, no double-blind trial had been carried out. Therefore, it is important to initiate research programs aimed towards the development of new medical countermeasures against CCHFV.

Since its discovery in 1998 [22], RNA interference (RNAi) has been successfully applied as a technology to inhibit gene expression. Small interfering RNAs (siRNAs), the mediators of RNAi, are a class of double-stranded RNA molecules (20-25 nucleotides in length) that interfere with translation by inducing sequence-specific degradation of homologous mRNA [23,24]. Recently, several RNAi-based applications for gene silencing have been developed to target pathogenic human viruses causing acute or chronic infections including HIV-1 [25,26], influenza virus [27,28], respiratory syncytial virus [29,30], hepatitis B [31-34] and C viruses [35-37], as well as Marburg and Ebola filoviruses [38-40].

In this report, we tested various chemically synthesized siRNAs for their ability to inhibit HAZV replication in cell culture. We demonstrated that siRNAs targeting the NP mRNA depicted a stronger antiviral effect than those designed to inhibit the L and M segment encoded mRNAs. siRNAs were efficient when transfected in cells before or after HAZV infection and their use in combination with ribavirin induced a synergistic or an additive antiviral effect, according to the dose of ribavirin used. Thus, our study highlights the potential of RNAi in the antiviral treatment of nairovirus infection.

## Methods

### Cell lines and viruses

A549 cells (human lung carcinoma cell line, ATCC CCL-185) and Vero E6 cells (African green monkey kidney, ATCC CRL-1586) were grown in F12K medium and Dulbecco's modified eagle medium DMEM (Gibco, Invitrogen Corporation, Paisley, United Kingdom), respectively, supplemented with 10% heat-inactivated fetal calf serum (FCS; Invitrogen, Sao Paulo, Brazil) and maintained at 37°C in a 5% CO_2 _atmosphere. BHK21 cells (baby hamster kidney, ATCC CCL10) were cultured in Glasgow minimum essential medium GMEM (Gibco, Invitrogen Corporation) with 10% FCS, 10% tryptose phosphate (Sigma-Aldrich, St Quentin-Fallavier, France) and 50 mM HEPES (Gibco, Invitrogen Corporation) at 37°C in a 5% CO_2 _atmosphere.

The HAZV strain JC280 used in these experiments was produced by infecting 90% confluent BHK21 cells at a multiplicity of infection (MOI) of 0.001. Virus was titrated using a focus-forming assay in Vero E6 cells as described below. Viral stocks usually reached 10^6^-10^7 ^foci forming units per ml (ffu/ml).

### HAZV titration

Monolayers of Vero E6 cultured in 12-well microplates were infected with serial 10-fold dilutions of supernatant from infected cells. After 1 hr incubation at 37°C, 3.2% carboxymethylcellulose (CMC) sodium salt (VWR International Ltd, Poole, England) were added into each well. CMC overlay was removed five days post-infection and cells were fixed with 4% formaldehyde for 20 min at room temperature (RT) and permeabilized in 0.5% Triton X-100 (Sigma-Aldrich, St Quentin-Fallavier, France) for 5 min. Viral foci were detected by probing with mouse anti-HAZV hyperimmune ascitic fluid (1:2000) for 1 hr at 37°C, followed by horseradish peroxydase-conjugated goat anti-mouse IgG (1:2000, Interchim, Montluçon, France) at 37°C for 1 hr. The cell monolayer was then incubated with 0.7 mg/ml of 3,3'-diaminobenzidine (DAB) solution (Sigma-Aldrich, St Quentin-Fallavier, France) diluted in PBS 1X for 10 min at RT. Once clusters of infected cells were visible (dark stain), the reaction was stopped by removing the DAB solution followed by water washing. The foci were counted manually under the light microscope.

### Design and synthesis of siRNAs

The sequences of HAZV L, M and S genomic segments [GenBank:DQ076419.1, DQ813514.1 and M86624.1, respectively] were used to design the siRNAs. Duplexes of 21-nucleotide siRNAs with short 3' overhangs were synthesized by Qiagen (Courtaboeuf, France). For each viral mRNAs, four lyophilized siRNAs were produced (Table 1) and their sequence was subjected to a BLAST search against GenBank to minimize off-target effects. In the present study, a non targeting siRNA (siNT) showing no complementarities neither with HAZV mRNAs nor with any human, mouse or rat mRNAs was used as a negative control (Table 1). The TOX siRNA (siTOX) (Dharmacon RNAi technologies, Lafayette, USA) was used to determine transfection efficacy (see below). Before use, all siRNAs were reconstituted in rehydration buffer to obtain 20 μM solutions according to the company's instructions.

**Table 1:** List of siRNAs used in this study

siRNA	Targeted genomic region	Sequence of duplex siRNA	GenBank accession number
siS1	91 to 111	*Sense *5'-AGAUUGUUGCCAGUACUAAdTdT-3' *Antisense *5'-UUAGUACUGGCAACAAUCUdTdG-3'	S Hazara segment JC280 [DQ076419.1]
siS2	1321 to 1341	*Sense *5'-AGGCAGUCCUCAACUAUAAdTdT-3' *Antisense *5'-UUAUAGUUGAGGACUGCCUdTdT-3'	
siS3	641 to 661	*Sense *5'-CGAUGAUGCGCCAAAGAGAdTdT-3' *Antisense *5'-UCUCUUUGGCGCAUCAUCGdGdA-3'	
siS4	881 to 901	*Sense *5'-CAAAGACCAAGUCGACCAAdTdT-3' *Antisense *5'-UUGGUCGACUUGGUCUUUGdTdT-3'	

siM1	3260 to 3280	*Sense *5'-CAGUCAUGAUGGUGGUUUAdTdT-3' *Antisense *5'-UAAACCACCAUCAUGACUGdTdG-3'	M Hazara segment JC280 [DQ813514.1]
siM2	3929 to 3949	*Sense *5'-CGAGCAUAAGGGUACAAUAdTdT-3' *Antisense *5'-UAUUGUACCCUUAUGCUCGdAdG-3'	
siM3	1689 to 1709	*Sense *5'-GAUGAAAGUUGCUCCUAUAdTdT-3' *Antisense *5'-UAUAGGAGCAACUUUCAUCdGdT-3'	
siM4	2924 to 2944	*Sense *5'-CAAAUACUUUGUCACCAAAdTdT-3' *Antisense *5'-UUUGGUGACAAAGUAUUUGdTdT-3'	

siL1	10545 to 10565	*Sense *5'-CGAGGAUAAUAUUGGCAAAdTdT-3' *Antisense *5'-UUUGCCAAUAUUAUCCUCGdTdG-3'	L Hazara segment JC280 [M86624.1]
siL2	9847 to 9867	*Sense *5'-CGAGGAUAAUAUUGGCAAAdTdT-3' *Antisense *5'-UAAAGUGUUUAUUAUAACCdTdG-3'	
siL3	1408 to 1428	*Sense *5'-CAGAGAAAUUGCUGAUAUAdTdT-3' *Antisense *5'-UAUAUCAGCAAUUUCUCUGdTdG-3'	
siL4	10323 to 10343	*Sense *5'-CAGUGAUAAAGGUAAAGAAdTdT-3' *Antisense *5'-UUCUUUACCUUUAUCACUGdGdG-3'	

Non targeting siRNA (siNT)		*Sense *5'-UUCUCCGAACGUGUCACGUdTdT-3' *Antisense *5'-ACGUGACACGUUCGGAGAAdTdT-3'	

### siRNAs transfection and A549 cells infection with HAZV

Twenty four hours before transfection, A549 cells were seeded in 24-well microplates at a density of 8 × 10^4 ^cells/well to achieve 60% confluent cell monolayers the day after. Various siRNAs concentrations (ranging from 0.01 to 100 nM) were complexed with the Lipofectamine 2000 transfection reagent (Invitrogen, Cergy Pontoise, France) in Opti-MEM I medium (Gibco, Invitrogen Corporation, Paisley, United Kingdom). The final volume of Lipofectamine 2000 was 1.5 μl/well. The transfection mixture was incubated for 20 min at RT to allow the formation of siRNA/transfection reagent complexes and 100 μl of the solution were added in each well. One day post-transfection, cells were gently washed twice with F12K medium and infected with HAZV at a MOI of 0.1. The inoculum was incubated for 1 hr. Cells were then cultivated in F12K medium supplemented with 0.4% FCS for 48 hrs. Infected cells supernatants were tittered as described above. The EC_50 _was calculated as the mean of two independent experiments using the GraphPadPrism version 4.00 software (GraphPad Software, San Diego California, USA) for non linear regression.

For the post infection treatment studies, 60% confluent A549 cell monolayers were infected with HAZV for 1 hr at a MOI of 0.01. At 1 hr, 8 hrs or 24 hrs post-infection, 100 μl of the transfection mixture (containing 100 nM of siRNA) were added. One day after transfection, cells were washed, and grown in F12K medium with 0.4% FCS for 48 hrs. The supernatant from infected cells were then harvested and tittered.

For each experiment, transfection efficiency was monitored by transfecting A549 cells with 100 nM of siTOX under the same experimental conditions as described above. Cells successfully transfected with siTOX undergo apoptosis and cell death within 24-48 hrs. After 3 days of incubation, siTOX-transfected cells were trypsinized and manually counted using a hematocytometer (Trypan blue exclusion assay). Transfection efficiency was calculated as the ratio between the number of viable siTOX-transfected cells versus non-transfected cells. In our experiments, transfection efficiency was routinely above 90%.

### Detection of HAZV nucleoprotein by Western blot

A549 cells, seeded in 6-well microplates at a density of 3.2 × 10^5 ^cells/well, were transfected with siRNA and infected with HAZV as described above. Protein extraction was performed 48 hrs post-infection as follow: confluent cells were washed twice in fresh phosphate-buffered saline 1X (PBS 1X) and lysed in buffer containing 20 mM Tris pH 7.5, 100 mM NaCl, 0.6% NP40, 0.5 mM EDTA and protease inhibitors cocktail (Complete EDTA-free, Roche Diagnostics GmbH, Mannheim, Germany) for 10 min on ice. After removal of cellular debris by centrifugation at 12,000 × g for 10 min, 20 μl of protein extracts were boiled for 5 min in Laemmli buffer and separated on a 10% SDS-PAGE. Proteins were then electrotransferred onto a polyvinylidene fluoride (PVDF) membrane (Bio-Rad Laboratories, Marne-la-Coquette, France). The PVDF membrane was saturated with 5% dry milk in PBS 1X containing 0.1% Tween-20 and incubated overnight at 4°C with mouse anti-HAZV hyperimmune ascitic fluid (1:250) or with mouse anti-GAPDH monoclonal antibody (1:1000, Ambion, Austin, TX, USA). Horseradish peroxydase-labeled goat anti-mouse IgG (1:10000, Interchim, Montluçon, France) was used as secondary antibody followed by chemoluminescent (ECL) revelation (Amersham GE Healthcare, Orsay, France).

### ELISA-based assay for interferon-β detection

A549 cells were cultured in 24-well microplates at a density of 8 × 10^4 ^cells/well and transfected as previously described with either 100 nM (i.e. 1.48 μg/ml) of siNT, siS1 and siS2 or 0.25 μg/ml of poly(I:C) dsRNA (Sigma-Aldrich, St Quentin-Fallavier, France). Cell culture supernatants were harvested 24 hrs post-transfection to detect human beta interferon (IFN-β). The cytokine measurement was performed using a sandwich enzyme-linked immunosorbent assay (ELISA) kit (PBL Biomedical Laboratories Tebu-Bio, Le Perray-en-Yvelines, France), according to the manufacturer's instructions.

### Antiviral assays with ribavirin and combination with siS1 or siS2

Confluent monolayers of A549 cells in 24-well plates were infected with HAZV at a MOI of 0.1. Cells were then cultivated with F12K medium supplemented with 0.4% FCS or treated with 0.4% FCS F12K medium containing serial dilutions of ribavirin (1-β-D-ribofuranosyl-1H-1,2,4-triazole-3-carboxamide) (Sigma-Aldrich, St Louis, Missouri, USA). Forty eight hours post-infection, the supernatant of each well was collected and virus titer was performed. The EC_50 _value for ribavirin was determined as the mean of two independent experiments.

The combination assay required 60% confluent monolayers of A549 cells in 24-well microplates transfected with siS1, siS2 or siNT at a concentration of 1 nM and 10 nM. One day post-transfection, cells were washed twice, infected with HAZV at a MOI of 0.1. One hour after infection, the inoculum was removed and transfected cells were cultured for 48 hrs in 0.4% FCS F12K medium containing 0, 25 or 50 μM of ribavirin. The cell supernatants were then tittered.

### Statistical analysis

In this study, we compared the antiviral effect of selected siRNAs to the negative control (siNT) to detect significant variations using the Student's t-test (P ≤ 0.05 was regarded as significant difference between the two groups of transfected/infected cells).

## Results

### Inhibition of HAZV replication using segment-specific siRNAs

**Figure 1:**
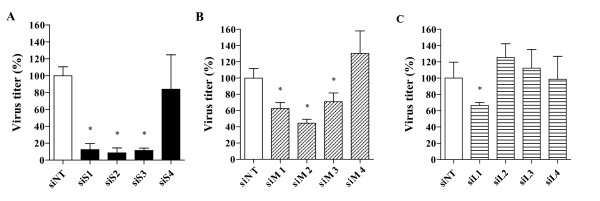
**Inhibition of HAZV replication by segment-specific siRNAs in A549 cells**. **A) **Cells were transfected with 100 nM of the different siS (siS1 to siS4), **B) **siM (siM1 to siM4) or **C) **siL (siL1 to siL4). Twenty four hours post-transfection, cells were infected with HAZV at a MOI of 0.1. Viral titers were determined 48 hrs post-infection as described in the materials and methods section. Results are expressed as a percentage of average foci counts in siRNAs treated cells to that in siNT (siRNA negative control) transfected cells. Errors bars represent the standard deviation (SD) of the means for at least two independent experiments performed in quadruplicates. * Significant differences compared to the siNT control: Student's t-test; *P *< 0.05.

To evaluate the inhibitory activity of siRNAs on HAZV replication, twelve siRNAs were designed to target the mRNAs produced by the L, M, and S genomic segments (Table 1). We analyzed the inhibitory effect of the L, M and S-specific siRNAs. As shown in figure 1A, when used at 100 nM, siS1, siS2 and siS3 strongly inhibited virus replication by 87.8%, 91.6% and 86.1%, respectively, compared to siNT control (p < 0.05). In contrast, siS4 did not induce any significant antiviral effect in A549-infected cells. siM1, siM2 and siM3 showed moderate but significant activities on HAZV replication (38.3%, 56.2% and 29.4% of inhibition respectively) whereas siM4 did not have any activity (figure 1B). Lastly, siL2, siL3 and siL4 did not inhibit HAZV replication (p > 0.05) while siL1 slightly did (33.5% of inhibition, p = 0.04, figure 1C).

### Inhibition of HAZV replication by siS1 and siS2 in A549 cells

**Figure 2:**
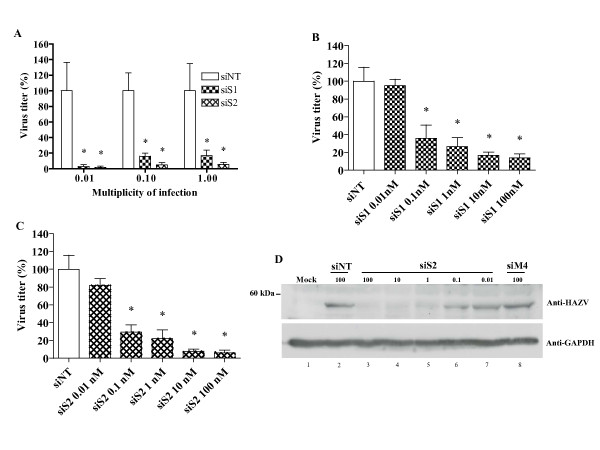
**Inhibition of HAZV replication with siS1 or siS2 in A549 cells**. **A) **Cells were transfected with 100 nM of siS1 or siS2 and then infected with HAZV at different MOI (i.e. 0.01, 0.1 and 1). **B) **and **C) **24 hrs before HAZV infection (at a MOI of 0.1) cells were transfected with different concentrations of siS1 or siS2 (from 0.01 to 100 nM). Viral titers were determined 48 hrs post-infection. Results are presented as a ratio between the average foci counts from siRNAs treated wells and counts in siNT (siRNA negative control) transfected cells. Errors bars represent the standard deviation (SD) of the means for at least two independent experiments carried out in quadruplicates. * Significant differences compared to the siNT control: Student's t-test; *P *< 0.05. **D) **Lysates from A549 cells infected with HAZV and treated with different concentrations of siS2 (or 100 nM of siNT or siM4) were loaded on a 10% SDS-PAGE and electrotransferred on a PVDF membrane. Expression of the ~ 50 kDa NP was detected with an anti-HAZV ascite (GAPDH expression was used as loading control).

Since siS1 and siS2 showed the most efficient anti HAZV activity, we decided to further focus on the characterization of these two siRNAs. We first tested their efficacy at 100 nM in cells infected at different multiplicity of infection (i.e. MOI 0.01, 0.1 and 1). Whatever the viral load, a significant inhibition of HAZV replication was observed for both siRNAs when compared to siNT: the inhibition ranged from 83.1% to 97.1% for siS1 and from 94.4% to 98.3% for siS2 (figure 2A).

Because siRNAs treatment could be cytotoxic and therefore may affect viral growth, we performed a Trypan blue exclusion assay to evaluate cell growth and viability upon siRNA transfection. The morphology of treated cells was examined daily using phase-contrast light microscopy. Three days post siRNA transfections, the cell number per well was determined and compared to non treated cells by manual counting with a hematocytometer. In these experiments, we did not detect any cytotoxic effect of siS1 and siS2 on A549 cells at any concentrations used (not shown). We also investigated whether the IFN pathway could be stimulated in siRNAs transfected cells as reported in earlier studies [41,42]. We found that the siRNAs tested did not induce any IFN-β response whereas the poly(I:C) control was a good stimulator (not shown). Thus, HAZV inhibition was siS1 and siS2 specific and was not due to any side effects.

We then examined the antiviral effects of increasing concentrations of siS1 and siS2. A concentration dependent inhibitory activity was observed for both siRNAs when compared to siNT (figures 2B and 2C). siS1 induced an average inhibition of 64.2% at 0.1 nM and 86% at 100 nM. The production of infectious HAZV particles was reduced by 70.6% at 0.1 nM and 94% at 100 nM when cells were transfected with siS2. No significant viral inhibition was observed at siRNAs concentration of 0.01 nM. From these experiments the estimated 50% effective concentration (EC_50_) was 0.09 nM for siS1 and 0.07 nM for siS2. Thus, both siRNAs efficiently inhibited HAZV replication in a concentration dependant manner, in cell culture.

### Specific inhibition of NP expression by siS1 and siS2

Since siS1 and siS2 were designed to target the NP transcript, we wanted to demonstrate that HAZV replication inhibition was associated with NP synthesis reduction. As shown in Figure 2D, when cells were transfected with different concentrations of siS2, a dose-response decrease of NP expression was observed. The ~50 kDa NP was hardly detectable in cells treated with 100, 10 and 1 nM of siS2 (lanes 3, 4 and 5). This was in sharp contrast with the amount of NP seen in siNT transfected-cells (lane 2). When siS2 was used at 0.1 nM, NP synthesis was moderately inhibited (lane 6) while no inhibition was observed in cells transfected with 0.01 nM of siS2 (lane 7). As expected, siM4, which did not inhibit HAZV replication (Figure 1B), did not interfere with NP expression (lane 8). A similar siS1 dose response inhibition of NP production was observed (not shown). Thus, the decrease of NP synthesis by siS1 and siS2 completely parallels the HAZV replication inhibition (Figures 2B and 2C).

### Antiviral activities of siS1 and siS2 transfected before and after HAZV infection

**Figure 3:**
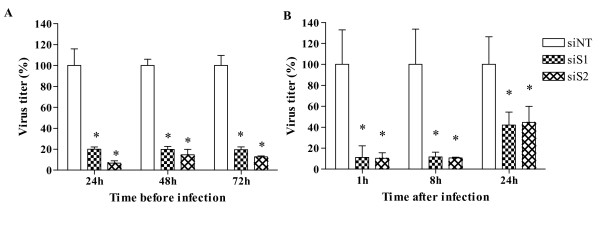
**Antiviral activities of siS1 and siS2 transfected before and after HAZV infection**. **A) **A549 cells were transfected with 100 nM of siNT (siRNA negative control), siS1 or siS2, 24 hrs, 48 hrs or 72 hrs before HAZV infection (MOI 0.1). **B) **Cells were infected with HAZV at a MOI of 0.01 and then transfected with 100 nM of siNT, siS1 or siS2, 1 hr, 8 hrs or 24 hrs post-infection. Viral titers were determined 48 hrs post-infection. Results are shown as a ratio between virus titer obtained in siRNAs treated wells and titer determined in siNT transfected cells. Errors bars represent the standard deviation (SD) of the means for at least two independent experiments done in quadruplicates. * Significant differences compared to the siNT control: Student's t-test; *P *< 0.05.

To estimate the stability of siRNA *in vitro*, we performed a time-course experiment in which A549 cells were infected at 24 hrs, 48 hrs or 72 hrs after siS1 or siS2 treatment (figure 3A). A significant reduction of HAZV replication was observed when infection occurred 24 hrs post-transfection (80% with siS1 and 93% with siS2). Interestingly, the antiviral effect was maintained at least three days post-treatment (up to 80.4% and 87.4% inhibition using siS1 and siS2 respectively), indicating the siRNAs stability over this period of time.

We also studied the antiviral effect of siS1 and siS2 transfected at various time post-infection (p-i). As illustrated in figure 3B, when cells are treated 1 hr or 8 hrs p-i, HAZV titer was reduced by more than 88%. When cell transfection was performed 24 hrs p-i, a moderate inhibition > 50% was still observed (P < 0.05). This last experiment indicated that when used at 100 nM both siRNAs exhibited a significant antiviral activity on HAZV that had already been replicating for 24 hrs.

### Additive or synergistic inhibition of HAZV replication by ribavirin and siS1 or siS2

**Figure 4:**
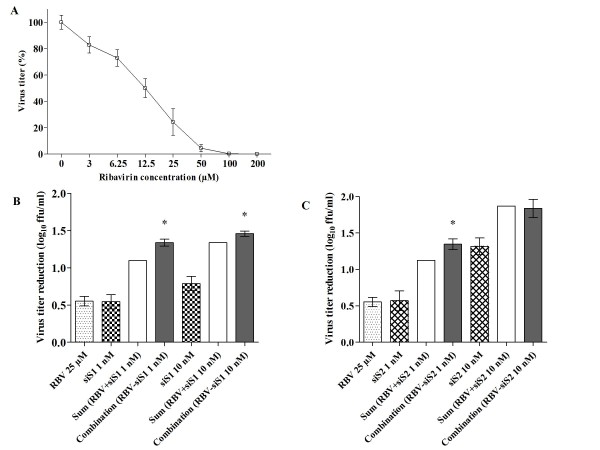
**Inhibitory effect of ribavirin and combination with siS1 and siS2**. **A) **A549 cells were infected with HAZV at a MOI of 0.1 and treated with different concentrations of ribavirin (ranging from 0 to 200 μM) for 48 hrs. Results are presented as a ratio between virus titer obtained in ribavirin treated wells and titer determined in non-treated cells. **B) **A549 cells were transfected with 1 nM or 10 nM of siS1 or **C) **with 1 nM or 10 nM siS2. Cells were infected with HAZV at a MOI of 0.1 and then incubated for two days in medium containing 25 μM of ribavirin. Viral titers were determined 48 hrs post-infection. The reduction of virus titer is determined after treatment with either ribavirin or siS1 (or siS2) and with the combination of ribavirin and siS1 or siS2. The white bar represents the theoretical sum of antiviral activity obtained with each compound. The synergistic effect of the combination of ribavirin and each siRNAs is indicated by the grey bar. Nb: no synergy was observed when siS2 was used at 10 nM with ribavirin. * Significant differences between additive and synergistic effect: Student's t-test; *P *< 0.05. Errors bars represent the standard deviation (SD) of the means for at least two independent experiments performed in quadruplicates.

As depicted in figure 4A, ribavirin, which is a ribosyl purine analogue, exhibited a clear concentration-dependent anti HAZV activity leading to a complete inhibition at 100 μM. No cytotoxic effect of ribavirin was observed in the range of concentrations used (data not shown). The ribavirin EC_50 _was estimated at 8.9 μM.

Since ribavirin and siRNAs (i.e. siS1 and siS2) have two distinct modes of action and target two different viral components (viral polymerase and mRNA coding for NP, respectively), we wondered whether the combination of ribavirin and siRNAs could induce a higher inhibitory activity than the sum of the individual activities. We performed an assay in which A549 cells were transfected with siS1 or siS2, 24 hrs before being infected with HAZV and then subsequently treated with 25 μM of ribavirin. As shown in Figure 4B, when cells were treated with either 1 nM or 10 nM of siS1 in combination with 25 μM of ribavirin, a significant synergistic antiviral activity was observed. Similarly, siS2 at 1 nM in association with 25 μM of ribavirin reduced the virus titer in a synergistic manner (P < 0.05) (figure 4C). However, at a concentration of 10 nM, the combination of siS2 with ribavirin only induced an additive effect (figure 4C). In the same way, when ribavirin was used at 50 μM, an additive antiviral activity was observed with 1 or 10 nM of siS1 or siS2 (data not shown). Thus, these experiments suggest that the combinatorial treatment including ribavirin and NP-targeting siRNAs could represent a strategy to control nairovirus infection.

## Discussion

With the noticeable exception of ribavirin recommended by the World Health Organization (WHO) to treat CCHFV infection [43], there is no specific medical therapy. Therefore, there is a need for the development of novel antiviral strategies against nairovirus infections.

In this work, for the first time, we evaluated the antiviral activity of siRNAs targeting the L (polymerase), M (glycoproteins) and S (nucleoprotein) transcripts of HAZV, a non pathogenic nairovirus in humans, which is considered as a surrogate CCHFV model.

We observed that siRNAs complementary to the mRNAs encoded by the L and M genomic segments had a lower effect than those targeting the S segment. Interestingly, three studies performed in arthropod cells showed that the nucleoprotein gene of orthobunyaviruses is a RNAi prim target. We demonstrated the efficient inhibition of HAZV in tick cells via RNAi induced by a Semliki Forest replicon expressing the S segment whereas the L segment had no effect [44]. In earlier studies, Billecocq *et al. *and Powers *et al. *observed a similar phenomenom in mosquito cells infected by Rift Valley Fever virus [45] and La Crosse virus [46], respectively. Furthermore, in mammalian cells La Crosse virus replication was successfully decreased by siRNAs targeting the S segment and similarly to our results, the L and M siRNAs had a much weaker effect [47]. Finally, orthobunyavirus Akabane replication was inhibited up to 99% by siRNAs directed against highly conserved regions of the nucleoprotein gene [48].

As other RNA viruses, nairovirus present a high mutation rate which might contribute to their escape from siRNAs inhibition. The emergence of viral mutants is lowered when using siRNAs targeting the most conserved viral sequences. Among the three genomic segments, the S segment is the most conserved within the *Nairovirus *genus because of its lowest mutation rate [49,50]. This observation indicates that siRNAs directed against the S segment are potentially interesting anti-nairovirus molecules.

The nucleoprotein plays a crucial role in the regulation of viral transcription and replication. It associates with genomic RNA and serves as a template for the polymerase to initiate both steps during virus cycle. It is assumed that cytoplasmic NP concentration is important for the transition from transcription to replication [2] and therefore, a decrease in NP production should contribute to virus inhibition, as observed in our study.

Among all tested siRNAs, only three (siS1, siS2 and siS3) exhibited a strong inhibition of HAZV replication (up to 90%). The unequal siRNAs efficiency is in agreement with previous works [51-53]. The interaction of proteins with the viral transcripts or the presence of secondary structures in these mRNAs may interfere the proper recognition by the homologous siRNAs [54,55], explaining the variability of siRNAs efficacy. Interestingly, the combination of siS1, siS2 and siS3 used at a concentration of 33 nM (100 nM siRNA final concentration) induced ~ 90% virus inhibition which is similar to the effect observed for each individual siRNAs (data not shown, figure 1A). The absence of additive or synergistic antiviral effect observed here was also reported in other studies. It was hypothesized that the competition of siRNAs for RISC might explain this lack of effect [56,57].

In our study, the two most active siRNAs, siS1 and siS2, demonstrated a specific inhibitory effect against HAZV in a dose-dependent manner. These two siRNAs did not induce IFN production or cell toxicity. We showed that treatment with each siRNAs correlates with the reduction of nucleoprotein expression level. A concentration as low as 0.1 nM for both siRNAs was sufficient to reduce significantly HAZV replication. A similar antiviral effect at such concentrations had already been described in a previous study with Epstein-Barr virus [58]. We observed that the siRNA stability lasted at least up to 72 hrs. The prolonged stability of siRNAs with a relatively long half life (from 3 to 8 days after transfection) was also shown in experiments with vaccinia virus [59], monkeypox virus [53], food and mouth disease virus [60] and HIV-1 [61]. We also demonstrated that the antiviral activity of siRNAs was effective when carried out after infection. Taken together, the results are encouraging for the use of siRNA as future prophylactic and therapeutic treatments of nairovirus infection.

Since its discovery [22], RNAi technology has been assessed in several clinical trials. Among these, the most developed assays include the treatment of HIV-1 [62] and RSV [63]. siRNAs were successfully used against viral diseases in numerous animal models including infections affecting the liver: hepatitits C virus [64,65], hepatitits B virus [31,33,34,66,67] or Hepatitis E virus [68]. These data suggest that a similar strategy may be considered to treat patients infected with CCHFV which has an important hepatotropism.

We demonstrated *in vitro *the inhibitory activity of ribavirin against HAZV in a concentration dependent manner. This guanosine analogue acts through several mechanisms including the replication of viral nucleic acid. Ribavirin and siRNAs having distinct modes of action and different target, it was tempting to use them in combination to improve the efficacy of each separate treatment. We reported here the additive or synergistic effect of siRNAs in combination with ribavirin without increasing the cellular toxicity. Our results highlight the interest of a combined therapy. Such a therapy is known to reduce the drug toxicity by decreasing the active dose [69] and preventing the antiviral compound resistance [70,71]. Ribavirin is a potent inhibitor of several viruses *in vitro *[16] and *in vivo *[18]. Thus, it should be interesting to associate ribavirin and siRNAs to treat CCHFV infections.

## Conclusions

We demonstrated the ability of siRNAs targeting transcripts encoded by S segment to inhibit Hazara nairovirus replication in mammalian cell cultures. We reported the strong antiviral effect of siRNA and ribavirin in a combined treatment. This promising strategy could be used in a future anti-nairovirus therapy.

## List of abbreviations

HAZV: Hazara virus; CCHFV: Crimean-Congo hemorrhagic fever virus; NSDV: Nairobi sheep disease virus; RNAi: RNA interference; siRNA: small interfering RNA; MOI: multiplicity of infection

## Competing interests

The authors declare that they have no competing interests.

## Authors' contributions

OF, JMC and FI participated in the conception and design of the study. OF performed most of the experiments. FI carried out the immunoblot assay. SV provided reagents and technical help. OF, CNP, MB, JMC and FI contributed to the analysis and interpretation of the data. OF, CNP, MB and FI wrote the paper. All authors read and approved the final manuscript.
